# Treatment of Tuberculous Meningitis and Its Complications in Adults

**DOI:** 10.1007/s11940-018-0490-9

**Published:** 2018-02-28

**Authors:** Angharad Davis, Graeme Meintjes, Robert J. Wilkinson

**Affiliations:** 1 National Hospital for Neurology and Neurosurgery, University College London Hospitals, London, WC1N 3BG UK; 2 University College London, Gower Street, London, WC1E 6BT UK; 3 Wellcome Centre for Infectious Diseases Research in Africa, Institute of Infectious Disease and Molecular Medicine and Department of Medicine, University of Cape Town, Observatory, Cape Town, 7925 Republic of South Africa; 4 The Francis Crick Institute, London, NW1 2AT UK; 5 Department of Medicine, Imperial College London, London, W2 1PG UK

**Keywords:** Tuberculous meningitis, Immunotherapies, Anti-tuberculous therapies, Hydrocephalus, Tuberculoma, Human immunodeficiency virus

## Abstract

**Purpose of review:**

Tuberculous meningitis (TBM) is a global health problem. In this review, we systematically evaluate the evidence for current and emerging antimicrobials, host-directed therapies and supportive managements.

**Recent findings:**

Current antimicrobial regimes do not factor the differing ability of drugs to cross the blood-brain barrier. Rifampicin may be more effective at higher doses yet the most recent clinical trial failed to demonstrate survival benefit at 15 mg/kg/day. Dose finding studies suggest that higher doses still may be safe and more effective. Fluoroquinolones are currently listed as important second-line agents in drug-resistant TBM; however, a survival benefit as a first-line agent has yet to be shown. Linezolid may be a promising antimicrobial with good central nervous system penetrance. Dexamethasone reduces mortality in HIV-uninfected individuals yet evidence for its use in HIV co-infection is lacking. Aspirin has anti-inflammatory and anti-thrombotic properties. Small studies have demonstrated efficacy in reducing stroke but further research is required to better understand its effect on controlling the host inflammatory response. Discovery of genetic polymorphisms may direct individualized immune therapies and mediators of the innate immune response may provide targets for the development of novel therapies. There is at present no significant evidence base to guide management of hydrocephalus in HIV co-infection.

**Summary:**

Further clinical trial data is required to improve treatment outcomes in TBM in particularly in regard to the value of high-dose rifampicin, newer antimicrobials with improved central nervous system penetration and host-directed therapies. Supportive measures in particular the management of hydrocephalus in HIV co-infection should be an area for future research.

## Introduction

Tuberculosis remains a major global health problem. In 2015, an estimated 10.4 million new cases of TB occurred worldwide. The World Health Organization’s ‘End TB Strategy’ calls for a 90% reduction in TB-related deaths and 80% reduction in TB incidence rate by 2030, 15 years on from its declaration. In 2015, the rate of reduction in yearly incidence was 1.5%, which falls below the required target of 4–5%. These figures reflect the ongoing evolving challenges faced in the prevention and treatment of tuberculosis [1].

In 1948, the modern era of tuberculosis treatment saw the first evidence of therapeutic response to streptomycin in pulmonary TB (PTB) [2]. Isoniazid followed in 1952 with a key trial demonstrating improved efficacy when added to streptomycin [3] and in 1971 the addition of rifampicin and pyrazinamide led to reduction in treatment duration from 2 years to 6 months [4]. However, unlike in PTB where decades of clinical trials have instructed and refined treatment regimens in drug-sensitive and more recently drug-resistant TB, comparatively, little evidence exists to guide optimal treatment in tuberculous meningitis.

Tuberculosis meningitis (TBM) is the deadliest form of tuberculosis with mortality highest in children [5] and HIV-1 co-infection (40%) [6]. The combined complications of HIV-1 co-infection and multi-drug resistance in TBM confer a mortality close to 100% [6]. Complications of TBM such as hydrocephalus and cerebral vasculitis add to the complexities of treatment.

In this review, we evaluate evidence guiding the treatment of TBM in adults. We consider three aspects to successful management: (i) effective antimicrobial treatments, (ii) controlling the host inflammatory response, and (iii) supportive interventions to reduce raised intracranial pressure. We discuss TBM complicated by HIV-1 co-infection in particular timing of antiretroviral therapy. We consider efficacy of current treatments, review the evidence for emerging therapies and suggest areas for future research.

### Antimicrobials

Current WHO guidelines for TBM are based on those developed to treat PTB and suggest treatment with 2 months of rifampicin (RMP), isoniazid (INH), pyrazinamide (PZE) and ethambutol (ETB) followed by up to 10 months of RMP and INH for all patients [7]. Although initiation of this regimen before the onset of coma is the strongest predictor of survival from TBM [8], this regimen does not take into account the differential ability of anti-tuberculosis drugs to penetrate the brain [9].

Introduced in 1952, INH made immediate impact on mortality in all forms of tuberculosis. This drug penetrates the CNS freely [10] and is a key chemotherapeutic agent in TBM with proven potent bactericidal activity [11]. RMP does not penetrate the blood-brain barrier (BBB) as well with concentrations in CSF only 10–20% of that reached in plasma [9]. This observation may not reflect the amount of ‘active’ RMP; in plasma, the majority of RMP is protein bound and only the unbound portion is active, whereas in CSF, very little is protein bound. The 10–20% of RMP noted in CSF may be comparable to levels of active drug in plasma. There has been some research into the efficacy of higher dose RMP in TBM [12••, 13, 14]. In a randomized study which informed the current recommended dose of RMP, patients with PTB received either 450 mg (7.5 mg/kg), 600 mg (10 mg/kg) or 750 mg (12.5 mg/kg) of RMP with a fixed dose of INH. There was no observed difference in rate of sputum conversion in those who received 750 mg OD and 600 mg OD; however, those who received 450 mg OD showed a lower rate of sputum conversion and a higher rate of treatment failure [15]. More recent in vitro, animal and early bactericidal activity studies suggest that the 600-mg once daily dose is at the lower end of the dose-response curve [16]. In 2013, an open-labelled randomized phase 2 study in 60 Indonesian adults with TBM showed a 50% reduction in mortality with high dose (600 mg, about 13 mg/kg) intravenously compared to standard oral dose (450 mg, about 10 mg/kg) RMP. A larger randomized placebo-controlled trial in Vietnam recruiting between 2011 and 2014 tested a higher dose of oral RMP alongside levofloxacin against standard therapy and failed to show a mortality benefit with an oral dose of 15 mg/kg [12••]. Around the same time, a dose ranging trial conducted in PTB showed that doses up to 35 mg/kg were safe and well tolerated in the first 2 weeks of therapy [17•]. In the same study, the highest doses of 30 and 35 mg/kg showed highest early bactericidal activity measured by fall in colony-forming units (CFU) and time to positivity (TTP). At 2 weeks, 8 out of 14 patients taking 35 mg/kg of RMP were culture negative compared 5 of 14 taking 20 mg/kg, 0 of 15 taking 25 mg/kg2 of 15 taking 30/kg and 3 of 8 controls [17•]. This data may suggest that the selected dose of 15 mg/kg was not high enough and provides impetus for evaluating higher doses of RMP in TBM.

Pyrazinamide (PZA) which was introduced simultaneous to RMP as a potential agent with a role in treatment shortening [18] has good CSF penetration; concentrations in CSF are close to that of serum [9]. Though PZA has poor bactericidal activity in the first 2–4 days of treatment, studies in PTB have shown that thereafter (days 4–14), its activity matches that of INH and RMP [19, 20].

Of the current recommended first-line drugs, penetration of ethambutol (ETB) is the poorest, even when the BBB is inflamed which raises the question of its value within the treatment regimen [9]. With this and the issue of preventing INH resistance in mind, debate has arisen regarding the choice of a fourth drug in treatment of drug-sensitive TBM. Further urgency has been conferred by the global rise in RIF-resistant (RR) and multi-drug resistant TB (MDR-TB). MDR-TB is by definition infection resistant to RMP and INH; global incidence is estimated at 480,000 cases per annum [21]. WHO guidelines for the treatment of RR- and MDR-TBM state that at least five effective drugs should be used initially including a fluoroquinolone and an injectable second-line agent and treatment should last 18–24 months [21]. Recommended core second-line agents and their CSF penetrance are listed in Table 1. In 2016, the WHO issued guidance on standardized shorter MDR-TB regimens which was based on observational data reporting treatment with seven drugs over 9–12 months in 14 countries worldwide. The new recommendation is expected to benefit the efforts to reduce MDR-TB worldwide although there is risk of worsening resistance (leading to extensively drug resistant TB) if the regimen is used inappropriately [22]. There is currently no evidence base to support use of this regimen in TBM.

**Table 1:** Anti-tuberculous drugs in tuberculous meningitis and drug-resistant tuberculosis

First-line drugs for treatment of drug sensitive TBM in adults			
Drug	WHO-recommended daily dose	WHO-recommended duration	CSF penetrance (CSF:plasma concentration)
Rifampicin	10 mg/kg (range 8–12 mg/kg); max 600 mg	12 months	10–20%
Isoniazid	5 mg/kg (range 4–6 mg/kg); max 300 mg	12 months	80–90%
Pyrazinamide	25 mg/kg (range 20–30 mg/kg)	2 months	90–100%
Ethambutol	15 mg/kg (range 15–20 mg/kg)	2 months	20–30%
Second-line drugs for treatment of TBM in adults			
Levofloxacin	10–15 mg/kg	Throughout treatment	70–80%
Moxifloxacin	400 mg	Throughout treatment	70–80%
Amikacin	15 mg/kg; max 1 g. IV or IM.	Intensive phase only	10–20%
Kanamycin	15 mg/kg; max 1 g. IV or IM.	Intensive phase only	10–20%
Capreomycin	15 mg/kg; max 1 g. IV or IM.	Intensive phase only	No data (probably very low)
Ethionamide or prothionamide	15–20 mg/kg; max 1 g.	Throughout treatment	80–90%
Cycloserine	10–15 mg/kg; max 1 g	Throughout treatment	80–90%
Linezolid	600 mg	Throughout treatment	30–70%
Other drugs used in treatment of multi-drug-resistant TB but of uncertain benefit in TBM			
Clofazimine	100 mg OD	No recommended duration	Limited data (probably low)
p-Aminosalicylic acid	200–300 mg/kg	No recommended duration	No data (probably very low)
Bedaquiline	Not determined	New drug. Limited availability.	Probably very low (but data from one patient only)
Delamanid	Not determined	New drug. Limited availability.	No data

Of the fluoroquinolones, ofloxacin was the first to be recognized as a potential effective treatment for tuberculosis [23]. In 2011, a randomized study in Vietnam investigated the pharmacokinetics and exposure-response relationships of three fluoroquinolones in TBM. A total of 61 patients were assigned to standard treatment alone or standard therapy in combination with either ciprofloxacin, levofloxacin or gatifloxacin for the first 60 days of treatment. Population pharmacokinetic models describing the disposition of fluoroquinolones in CSF and plasma were used to determine exposure-response relationships through univariable analysis of clinical outcomes. These revealed consistent U-shaped exposure-response relationships for dichotomous and time to event outcomes. Significant higher proportions of death and disability were observed for patients with lower or higher plasma and CSF fluoroquinolone exposures than for patients with intermediate exposures. These findings may be explained by the increased permeability of the BBB in severe meningeal disease. It may also be a result of reduced creatinine clearance in those with more severe systemic involvement and therefore those more likely to die. Nonetheless, the study demonstrated improved clinical outcomes measured by survival, burden disability and incidence disease relapse of fluoroquinolones when used prior to the onset of comas and informs dose finding for future studies to test efficacy of fluoroquinolones in TBM [24]. In PTB, several studies have a higher rate of sputum conversion at 2 months of treatment with regimens containing fluoroquinolones; however, more adverse events and less favourable clinical outcomes have also been observed [25]. In PTB, the addition of a fluoroquinolone to standard treatment did not permit effective treatment shortening [26]. Since 2011, two studies in TBM (both discussed above) have evaluated the efficacy of adding fluoroquinolones to standard treatment in reducing mortality [12••, 27]. In a randomized double-blind study, a total of 817 adults with TBM received either standard therapy or an intensified regimen including a higher dose of RMP (15 mg/kg) as well as levofloxacin 20 mg/kg. This study failed to show a benefit of adding levofloxacin in TBM [12••]. The second study was a randomized controlled trial undertaken in Indonesia where 60 adults with TBM were randomized to receive standard dose (450 mg) or high dose (600 mg) RMP with either moxifloxacin (400 mg) or ethambutol (750 mg). This analysis demonstrated no relationship between exposure to moxifloxacin in plasma and CSF and survival [4]. Fluoroquinolones are currently listed as core second-line agents for the treatment of RR and MDR TBM; however, further large-scale phase 3 studies are required to address the question of survival benefit in TBM.

Linezolid, a synthetic antimicrobial and the first agent of the oxazolidinone class, was licensed in 2000 for treatment of nosocomial pneumonia and skin infections caused by select gram-positive bacteria [28, 29]. The role of linezolid in tuberculosis was first investigated in the context of MDR tuberculosis. Early studies reported rapid sterilization of *Mycobacterium tuberculosis* cultures following the administration of linezolid 600 mg BD in addition to standard treatment [30–32]. Following this, a number of studies demonstrated a role for linezolid as an effective treatment in drug-resistant tuberculosis [33–38]. There may be further benefit in patients with additional resistance to fluoroquinolones and with extensively drug-resistant TB [39]. In a systemic review and meta-analysis conducted to assess efficacy, safety and tolerability of linezolid in drug-resistant PTB, 15 studies including one randomized controlled trial were identified covering 367 patients of which 239 could be evaluated for effectiveness. Eighty-three percent [95% confidence interval, 75–90%) of those treated with linezolid had a favorable outcome which was defined as either treatment cure or completion [40]. A prospective open-label phase II to address the efficacy of linezolid as a substitute for ethambutol in drug-sensitive PTB is underway in China [41]. In TBM, evidence to support the use of LZD is limited. An observational study by Li et al. demonstrated favorable clinical outcomes and a non-significant difference in adverse events in children with drug-sensitive TBM treated with linezolid compared to control [42]. However, the study was a retrospective observational analysis with unblinded assessment of clinical outcomes. Although promising, further evidence is required to support its use in children. In adult TBM, there is similar paucity of data; in a retrospective cohort study of 33 adults with TBM, the addition of linezolid to a standard regimen led to more rapid improvement in CSF parameters, recovery of consciousness and reduction of fever. The study did not demonstrate a survival benefit but suggests a possible role for linezolid in adults with severe TBM [43•]. There has been concern regarding safety of linezolid in particularly given serious adverse events associated with its use such as bone marrow suppression, peripheral neuropathy and optic neuropathy. In the aforementioned systemic review, the most common adverse events were peripheral neuropathy (31%) and anaemia (25%). In the single RCT included within this meta-analysis, anaemia developed mainly during the first 4 months of treatment, whereas neuropathy developed after a longer course of treatment (5 months or more) [40].

Bedaquiline became available for the treatment of MDR-TB in 2013. It is the first antimycobacterial of the diarylquinoline group and has been shown to have good early bactericidal activity in PTB [44]. In TBM, a pharmacokinetic study of a single patient suggests CSF penetrance may be poor [45]. Nonetheless, others have hypothesized that this medication has a potential role in TBM and warrants further investigation [46].

### Host-directed therapies

In TB, there has been much recent interest in adjunctive host-directed immune interventions to either enhance protective immunity or regulate pathological tissue-damaging immunity [47]. Corticosteroids are the most widely used and researched host-directed therapy. A number of studies have determined efficacy of corticosteroids in TB since their use was first suggested in the 1950s [48–55, 56••]. The largest of these in Vietnam demonstrated that adjunctive corticosteroids improved survival in adults and adolescents with TBM [56••]. The most recent Cochrane review of all published trials of dexamethasone as an adjunctive treatment in TBM also demonstrated a survival benefit, but failed to demonstrate an effect on long term morbidity [8]. Both this trial and the Cochrane review highlight the lack of data that conclusively demonstrates a survival benefit from corticosteroids where TBM is associated with HIV-1. Although not powered to address the question of the efficacy of dexamethasone in HIV-1-associated TBM, there were 98 HIV-1-infected participants within the Vietnam trial. Within this subgroup, there was no significant effect of dexamethasone on the combined endpoint of death and disability or on death alone (stratified relative risk of death 0.78; 95% CI 0.59 to 1.04; *p* = 0.08). A larger study is underway to address this.

A focus of recent research is the identification of genetic polymorphisms in immune response genes, in particular a single polymorphism in the leukotriene A4 hydrolase (LTA4H) promotor which plays a role in the balance of pro-inflammatory and anti-inflammatory eicosanoids, thereby influencing expression of TNF alpha [57]. Studies in zebrafish and subsequently in humans have shown that expression of LTA4H can determine susceptibility to disease as well as response to corticosteroids [58]. In a retrospective analysis of patients enrolled to a trial of adjunctive dexamethasone in TBM, survival benefit was restricted to homozygotes with a TT genotype of the LTA4H (hyperinflammatory) in contrast to CC (hypoinflammatory) genotypes where dexamethasone was associated with harm [58]. More recently in an analysis of patients enrolled to a study of intensified antituberculous regimens and adjunctive dexamethasone, LTA4H genotype predicted survival in HIV-1-uninfected patients with the TT genotype patients significantly more likely to survive than those with the CC genotype. In this study, patients with the LT14H TT genotype had high pro-inflammatory cytokine concentrations (IL-1B, IL-1 and IL-6). However, those with CT and CC genotypes had intermediate or lower concentrations respectively. This may suggest that the suppression of inflammation by dexamethasone leads to survival benefit in patients with the TT genotype, however may be non-beneficial or even harmful in those with CT or CC genotypes [59]. This highlights the potential role for individualized immunotherapy where adjunctive corticosteroids are given on the basis of pre-treatment genotyping. Further work to explore this hypothesis in randomized controlled trials is required.

Improved understanding of immunopathogenesis in TBM has led to discovery of target sites for immunotherapies. The cytokine TNF alpha has been a target in both animal and human studies. In a rabbit model of TBM, inhibition of TNF-alpha by use of thalidomide resulted in survival benefit [60]. In a safety and tolerability study using thalidomide at escalating doses, thalidomide was safe and well tolerated as an adjunctive therapy to treat children with stage 2 TBM [61]. Clinical and radiological data also suggested improved outcome. The results of this study supported a phase 3 randomised controlled trial in paediatric TBM to test thalidomide against placebo in stage 2 and 3 disease. Thalidomide was given at a dose of 28 mg/kg/day for the first 28 days of treatment. Forty-seven children were enrolled, of which 30 received thalidomide. This study was terminated early as all adverse events and deaths occurred in the thalidomide arm. Debate around the influence of the high dose and late stage of disease on the adverse outcomes remains [62]. No further studies have taken place to investigate what was once a promising treatment option in paediatric TBM; however, recent data suggests thalidomide and TNF-alpha blockade may still have a role in tuberculous mass lesions where treatment with corticosteroids has failed [63].

Early (within 2–4 weeks of commencing anti-tubercular therapy) antiretroviral (ART) therapy of HIV-1-associated tuberculosis is associated with survival benefit in patients with low CD4 counts [64–66]. A meta-analysis including 8 randomised controlled trials in PTB compared survival in patients in whom antiretroviral therapy was started within 1–4 vs 8–12 weeks. Results demonstrated a survival benefit in patients newly diagnosed with tuberculosis and a CD4 count of less than 50/mm^3^ where antiretroviral therapy was commenced within 1–4 weeks of diagnosis [67]. However, initiating such otherwise life-preserving therapy early during TB treatment may be complicated by more frequent HIV-1-TB-associated immune reconstitution inflammatory syndrome (TB-IRIS). In the CNS, TBM-IRIS may be markedly inflammatory and associates with significant mortality [68]. In the aforementioned meta-analysis, there was a twofold increase in TB-associated IRIS in patients treated early with antiretroviral therapy. This feature, together with the lack of survival benefit observed in HIV-TBM patients prescribed early rather than deferred ART [69], has led to recommendation of a more conservative approach to the introduction of ART in HIV-1 TBM deferring until 4–6 weeks of anti-tubercular therapy. To our knowledge, no research has addressed the question as to whether the incidence or natural history of HIV-associated TBM is changing or has changed in light of more effective anti-HIV therapies of modern times; however, we feel this would be an important topic for future research.

Other than corticosteroids, thalidomide and antiretroviral therapies, there is little clinical trial data to support efficacy of other host-directed therapies in TBM. Aspirin has a possible anti-inflammatory as well as anti-thrombotic role in TBM which is discussed later in this review. As we understand more of the immune signaling pathways in the host’s response to tuberculosis, potential therapies continue to emerge. Infliximab is a TNF-alpha inhibitor which is FDA approved for use in inflammatory bowel disease, rheumatoid arthritis and some seronegative arthropathies. Although most reports of infliximab in TB relate to the reactivation of latent tuberculosis, there are some case reports where corticosteroid has failed to control inflammation yet subsequent reintroduction of infliximab has led to a near complete resolution of symptoms [70, 71]. Other therapies to consider include interleukin receptor 1 inhibitors anakinra (IL-1 alpha and beta) and canakinumab (IL-1 beta only). The study of TBM-IRIS provides insight into the inflammatory pathology of TBM. For instance, an unbiased whole-genome transcriptomic analysis of peripheral blood has highlighted the role of the innate immune response demonstrating increased expression of canonical and non-canonical inflammasome genes in those developing TBM-IRIS compared to non-IRIS controls [72•]. This observation provides support for a dominant role of the innate immune response in TBM inflammation and suggests novel targets for immunotherapies.

### Supportive therapies

Rich and McCordock were the first to describe the pathogenic mechanisms which lead to central nervous system tuberculosis [73]. Research since then has enabled better understanding of the natural history including the neurological sequelae such as hydrocephalus, vasculitis leading the cerebral infarction and metabolic abnormalities especially hyponatremia. Early recognition and management of these phenomena remains integral to the treatment of patients with TBM.

#### Hydrocephalus and raised intracranial pressure

The inflammatory infiltrate within the subarachnoid space or the ventricular pathways may lead to disruption of CSF flow resulting in hydrocephalus. Hydrocephalus can be communicating (caused by abnormal flow through the basal cisterns) or non-communicating (usually a later complication due to obstruction at the level of the fourth ventricle). Communicating hydrocephalus is more common and can be managed medically however may require intervention if progressing. Non-communicating hydrocephalus required rapid intervention. CSF diversion techniques such as ventriculoperitoneal shunts (VPS) and endoscopic third ventriculostomy are the mainstay of surgical treatment for hydrocephalus [74]. Evidence as to which technique is most effective is lacking.

A systematic review of 1038 adults and children with TBM and hydrocephalus demonstrated good outcome, defined as Glasgow Outcome Scale 4 or 5 (Table 2) in 58.2% of patients. Good outcomes were observed in more patients with less severe disease specifically those found to be Grade I (78.57%) and II (65%) compared to those with more severe (Grade IV disease) where only 31.5% survived (Table 2). Subgroup analysis demonstrated that good outcomes occurred in significantly fewer patients with HIV-1-associated TBM with only 25% patients of patients achieving a good outcome compared to 61% of HIV-negative patients [77]. In a study of 30 HIV-1-infected patients with TBM and hydrocephalus, participants underwent VP shunt placement and outcomes were compared to age- and gender-matched HIV-negative controls. Patients were followed up at two time points, discharge (short term) and 3 months after VP shunt insertion (long term). Although short-term outcomes were only marginally better in the HIV-negative group, long-term outcomes differed significantly with 66.7% mortality and 76.2% poor outcome in HIV-positive patients compared to 30.8% mortality and 34.6% poor outcome in the HIV-negative controls. Their study demonstrated that HIV seropositivity is an independent predictor of poor outcome, although they did identify that in patients with less severe disease at presentation, 80% had good outcomes. By contrast to previous studies, these results suggest a role of VP shunting in HIV-associated TBM in patients with less severe disease [78]. In paediatric TBM, a recent study showed that there is an association between the severity of hydrocephalus and CSF immune biomarkers GFAP and S100B [79]. It remains unclear as to whether this is due to the secondary compressive effect on brain parenchyma or whether these inflammatory mediators are involved in the pathogenesis of hydrocephalus. Further research is required to establish best evidence-based practice for the treatment of this common complication in TBM in particular for HIV-associated disease.

**Table 2:** Clinical rating scores in TBM

Glasgow outcome scale [75]		
1	Death	Severe injury or death without recovery of consciousness
2	Persistent vegetative state	Severe damage with prolonged state of unresponsiveness and a lack of higher mental functions
3	Severe disability	Severe injury with permanent need for help with daily living
4	Moderate disability	No need for assistance in everyday life, employment is possible but may require special equipment
5	Low disability	Light damage with minor neurological and psychological deficits
Modified Vellore grading scale for TBM-induced hydrocephalus [76]		
Grade	Glasgow Coma Scale	Clinical features
I	15	Headache, vomiting +/− neck stiffness No neurological deficit
II	15	Neurological deficit present
III	9–14	Neurological deficit may or may not be present
IV	3–8	Neurological deficit may or may not be present

Although hydrocephalus is the most common cause of raised ICP, elevated ICP can also be caused by other pathological processes within the CNS. In TBM, meningeal pathology may extend into the parenchyma and lead to encephalitis, whilst obliterative vasculitis within the vessels leads to infarction. These processes may result in cytotoxic and vasogenic oedema. The presence of parenchymal pathology may lead to failure of cerebral vascular autoregulation. Metabolic abnormalities such as hyponatraemia, hyperthermia and hypercapnia can cause further dysregulation. Thus, clinical management should be directed at the frequent monitoring and correction of abnormalities in gas exchange and tissue oxygenation, through mechanical ventilation (if necessary), meticulous fluid and electrolyte management, monitoring and intervention to treat raised intracranial pressure where appropriate as well as adequate temperature control. When there is no surgical intervention indicated, yet ICP remains high, hyperosmolar agents most commonly mannitol may be effective yet a randomised control trial to examine this theory is required [80, 81].

#### Hyponatraemia

Hyponatraemia defined as a plasma sodium level < 135 mmol/L occurs in 40–50% of patients with TBM [82]. Several mechanisms exist. Cerebral salt wasting (CSW) is characterised by natriuresis, hyponatremia and volume contraction in response to brain injury [83]. The syndrome of inappropriate anti-diuretic hormone (SIADH) is also associated with brain injury and occurs due to excessive release of antidiuretic hormone from the posterior pituitary gland resulting in inappropriate, continued secretion or action of the antidiuretic hormone arginine vasopressin (AVP) despite normal or increased plasma volume leading to hyponatraemia [84]. In a prospective hospital-based study conducted in India, of 76 patients with TBM, 34 (44.7%) had hyponatremia due to CSW in 17, SIADH in 3 and miscellaneous causes in 14 [82]. Distinguishing between CSW and SIADH is critical: their presentations are similar, but management is drastically different. By convention, SIADH is managed by fluid restriction and cerebral salt wasting by fluid administration. Some suggest that both conditions can be treated with hypertonic saline [83], whereas others state that fluid restriction, the traditional treatment for SIADH, has had little benefit in meningitis and might result in worsening hypovolaemia and harm [84]. This complex and often overlooked complication in TBM should be further investigated to define optimal investigation and management.

#### Tuberculomas

Tuberculomas can occur together with or independently of TBM. Clinical presentation depends on site and includes seizures, focal neurological weakness or symptoms of raised intracranial pressure due to hydrocephalus or mass effect. Tuberculomas commonly present as a feature of paradoxical worsening in patients treated for TB or in HIV-1-infected patients starting ART. In a randomized study to assess effect of dexamethasone on TBM-related cerebral MRI changes in Vietnam, 43 patients receiving either dexamethasone (*n* = 24) or placebo (*n* = 19) underwent serial MRI scans. The number of patients with one or more tuberculomas rose from 64% (14 of 22) before treatment to 74% (20 of 27) after 60 days. There was no effect of dexamethasone on incidence of tuberculoma formation or on resolution of tuberculomas [85]. The mainstay of treatment remains anti-tuberculous therapy, the duration of which is debated. There is lack of evidence in this area but consensus suggests four anti-tuberculous drugs for 18 months or until the tuberculoma resolves [7]. In some cases, where there is diagnostic doubt or where the size and anatomical location of the tuberculoma is causing clinical worsening, surgical excision may be required. Stereotactic craniotomy and excision of superficial small tuberculomas and microsurgery are procedures now used. In cases where there is no response to dexamethasone, alternative anti-inflammatory agents have been tried, particularly when the tuberculoma involves the optic chiasm and threatens vision. Some case series suggest that thalidomide could help to alleviate these problems [86–88]. Case reports have reported success with anti-TNF biological agents (such as infliximab) [70] and IFN-γ treatment [71].

#### Vasculitis and stroke

**Fig. 1:**
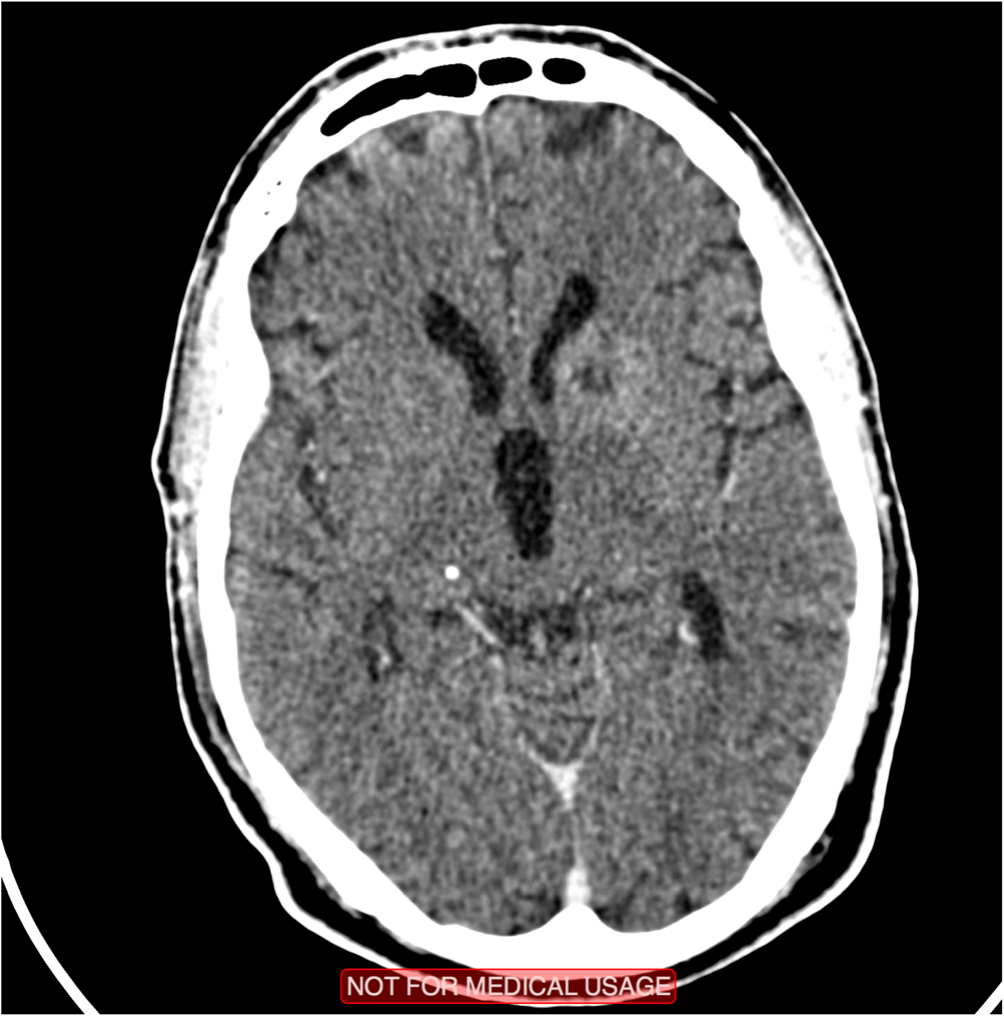
Computerised tomography (CT) of the head with evidence of probable perforator territory infarction on the left in an HIV-1-infected patient with tuberculous meningitis.

Stroke in TBM occurs in 15–57% of patients with TBM depending on the imaging modality used: CT reveals stroke in 13–35% [89, 90], MRI in 57% [91, 92]. They are usually multiple, bilateral and occur most commonly in deep grey matter structures including the caudate, anterior thalamus, anterior and genu of the internal capsule, namely the ‘tubercular zone’ [93] (see Fig. **1**). The macroscopic pathological appearance in the brain vasculature is that of gelatinous fibrocellular leptomeningeal infiltrates initially enveloping the vessels including the carotid arteries, middle cerebral arteries and their branches. Vasculitis within the affected vessels may occur with intimal proliferation. This process with or without superadded thrombosis leads to cerebral infarction [89]. There is no established prevention or treatment for stroke in TBM. Corticosteroids do not prevent stroke [56]. Aspirin has antiplatelet, anti-aggregant, anti-inflammatory and antioxidant properties [94, 95]. In a study of zebrafish, models with the hyperinflammatory LTA4H phenotype treated with aspirin showed reduced expression of pro-inflammatory eicosanoids and TNF alpha with subsequent modulation of inflammatory response [96]. In a placebo-controlled trial of aspirin in 118 adult patients with TBM in India, 150 mg OD aspirin was associated with a significantly lower 3-month mortality and a lower incidence of stroke that was not significant [97]. These findings suggest the effect of aspirin may be as much anti-inflammatory as anti-thrombotic. Following this, a similar study in South Africa randomized 146 children with TBM to receive low-dose (75 mg/24 h) (*n* = 47) or high-dose (1000 mg/24 h) aspirin. In this trial, there was no significant effect of aspirin on mortality; however, there was a significant effect on incidence of new hemiplegia in those receiving high-dose aspirin [98]. Although more effective therapies are now available for acute treatment and secondary prevention of ischaemic stroke associated with non-infectious vascular risk factors, aspirin an inexpensive well-tolerated drug remains the most commonly used treatment worldwide. Further large-scale randomized controlled trials are required to explore its role in reducing inflammation and vascular complications in TBM.

### Conclusions and research priorities

**Fig. 2:**
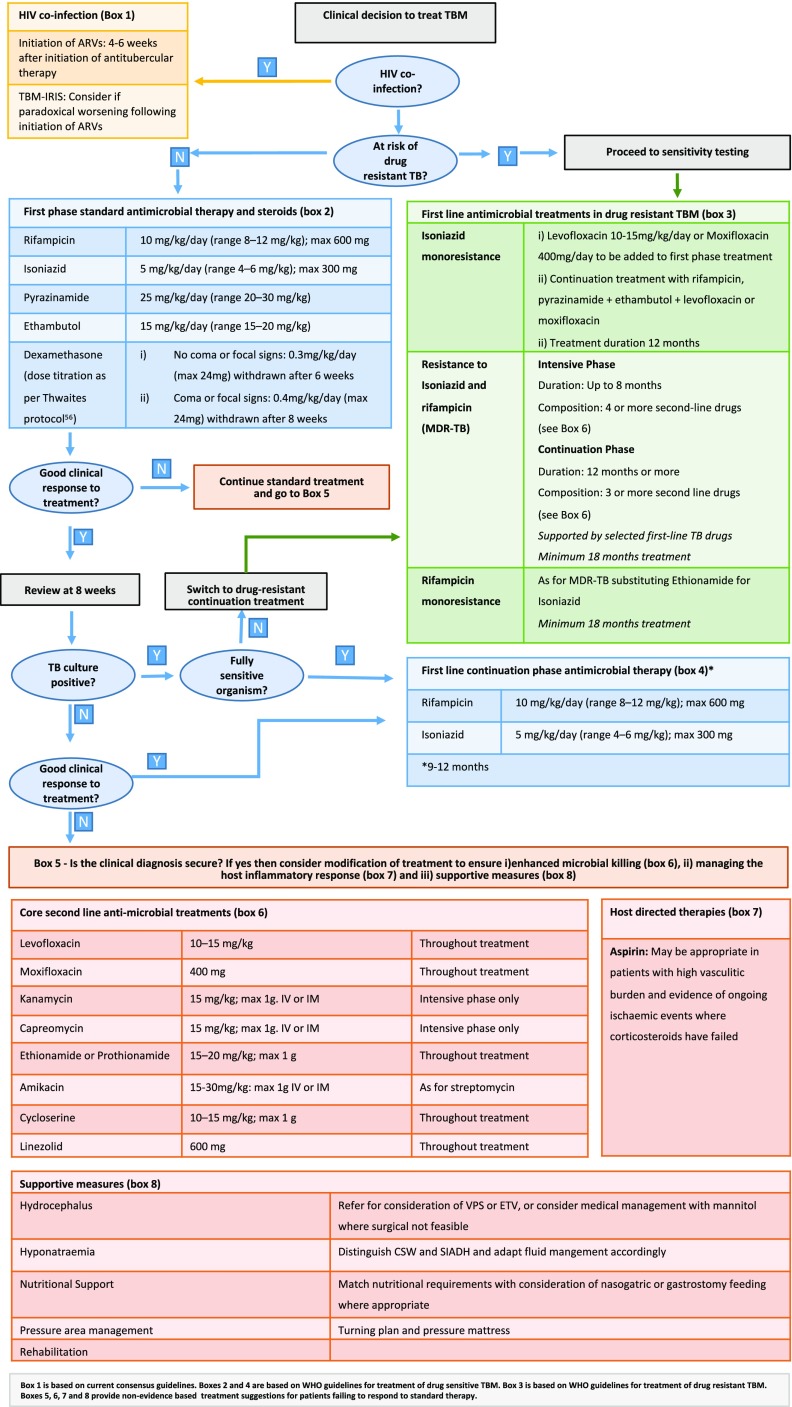
Treatment algorithm for patients with tuberculous meningitis.

- The neurological presentations of tuberculosis are the most lethal and under-researched manifestations of TB which remains a major global health problem.
- Current anti-tuberculous therapy regimens for TBM are based on those which are efficacious in PTB but do not consider the differing efficacy of drugs across the BBB. Further research is required to investigate the safety and efficacy of intensified therapy regimens and newer anti-tuberculous agents to treat CNS tuberculosis.
- Corticosteroids have proven mortality benefit except in HIV-associated TBM where as yet no sufficiently powered study has been able to prove benefit or lack thereof. More research is required to develop and evaluate novel host-directed therapies. Immune response phenotypes and genetic polymorphisms may direct individualized immune therapies and mediators of the innate immune response may provide targets for the development novel therapies.
- Stroke is a major cause of morbidity and mortality in TBM. Recent studies have shown a potential benefit of aspirin in the prevention of stroke as well as in the modulation of the host immune response in TBM. This requires further investigation in large phase 3 clinical trials.
- There is currently no significant evidence base to guide management of hydrocephalus in HIV-1-infected TBM. A large randomized clinical trial is required to investigate outcomes comparing available CSF diversion techniques in this particularly vulnerable subgroup of patients.
- A treatment algorithm provided in this paper gives a practical holistic approach to the management of patients with tuberculous meningitis (Fig. 2).
